# Transference of Traditional Versus Complex Strength and Power Training to Sprint Performance

**DOI:** 10.2478/hukin-2014-0054

**Published:** 2014-07-08

**Authors:** Irineu Loturco, Valmor Tricoli, Hamilton Roschel, Fabio Yuzo Nakamura, Cesar Cavinato Cal Abad, Ronaldo Kobal, Saulo Gil, Juan José González-Badillo

**Affiliations:** 1 NAR - Nucleus of High Performance in Sport, São Paulo, SP, Brazil.; 2 Pablo de Olavide University, Faculty of Sport, Seville, Spain.; 3 School of Physical Education and Sport, University of São Paulo, São Paulo, SP, Brazil.; 4 State University of Londrina, Londrina, PR, Brazil.

**Keywords:** transfer effect, complex training, traditional training, sprint speed performance

## Abstract

The purpose of this study was to determine the effects of two different strength-power training models on sprint performance. Forty-eight soldiers of the Brazilian brigade of special operations with at least one year of army training experience were divided into a control group (CG: n = 15, age: 20.2 ± 0.7 years, body height: 1.74 ± 0.06 m, and body mass: 66.7 ± 9.8 kg), a traditional training group (TT: n = 18, age: 20.1 ± 0.7 years, body height: 1.71 ± 0.05 m, and body mass: 64.2 ± 4.7 kg), and a complex training group (CT: n = 15, age: 20.3 ± 0.8 years, body height: 1.71 ± 0.07 m; and body mass: 64.0 ± 8.8 kg). Maximum strength (25% and 26%), CMJ height (36% and 39%), mean power (30% and 35%) and mean propulsive power (22% and 28%) in the loaded jump squat exercise, and 20-m sprint speed (16% and 14%) increased significantly (p≤0.05) following the TT and CT, respectively. However, the transfer effect coefficients (TEC) of strength and power performances to 20-m sprint performance following the TT were greater than the CT throughout the 9-week training period. Our data suggest that TT is more effective than CT to improve sprint performance in moderately trained subjects.

## Introduction

Improving sprint ability is critical for optimal performance in many sport disciplines. In this regard, a wide variety of training methods have been used such as speed training, plyometrics, resisted sprinting drills and traditional strength training (Cronin et al., 2005; de Villarreal et al., 2008; Saez de Villarreal et al., 2012).

Heavy resistance training (HRT), jump squats (Engelen-van Melick et al.), and countermovement jumps (CMJ) have been widely used by coaches to increase sprinting speed in professional athletes (McBride et al., 2002; Kraemer et al., 2003). The rationale behind the use of these methods is their efficacy to develop power and strength and the high correlation between these abilities and sprint performance (Alemdaroglu, 2012). For instance, Cronin et al. (2007) described that large increases in strength (i.e., maximum strength in squat exercise) are required in order to produce improvements in sprinting speed in recreational athletes.

In an attempt to maximize strength gains, a number of mixed training models has been suggested. Combined programs involving HRT, JS and CMJ are more effective at improving both maximum strength and power than isolated training methods (Adams et al., 1992; Cormie et al., 2007; Saez de Villarreal et al., 2013). However, the ideal combination of these methods during a training period still remains inconclusive.

In the traditional model of strength training (TT), a strength foundation phase is applied in the beginning of the macrocycle followed by a power phase (Hasegawa et al., 2002). Thus, HRT should be followed by progressive lighter resistances and higher velocity training loads. More recently, mixed training models such as “complex training” (Chodzko-Zajko et al.) were suggested to maximize performance associated with strength and power development (Duthie et al., 2002; Ebben, 2002). During CT, strength, power and plyometrics exercises are performed in the same training session. The use of the CT model is based on a classic exercise physiology theory in which the summation of the acute effects produced by each training unit determines the magnitude of the chronic adaptations (Bird et al., 2005; Mihalik et al., 2008).

Despite the well-established effects of the different training models on strength and power development and sprint ability improvements (Behrens and Simonson, 2011; Chelly et al., 2009; Maio Alves et al, 2010), no study has attempted to determine if any of these models is more effective to transfer strength and power increments to sprinting speed. In the aforementioned studies, subjects performed various type of sprint training (e.g. sprinting during matches or technical training sessions) alongside strength and power training. Therefore, it cannot be confirmed whether the increases in sprint performance were directly related to the strength and power gains.

According to Zatsiorsky and Kraemer (2006), the calculation of the “transfer effect coefficient” (TEC) should be used only when the subjects were not exposed to the target ability (i.e. sprint) during the strength/power training period. To express the magnitude of the TEC, the authors suggested an equation that represents “a ratio of resulted gains”. The resulted gain, also known as the effect size (ES) for a group is computed as follows:

$$\begin{array}{l} \text{ES}=(\text{Post}-\text{training}\;\text{mean}-\text{Pre}-\text{training}\\ \;\text{mean})/\text{Pre}-\text{training}\;\text{standard}\;\text{deviation} \end{array}$$

Thus, for the calculation of the TEC, a ratio between the resulted gains (ES) in the performed exercise and in the unperformed exercise is employed. The higher the ratio, the greater the TEC of the performed exercise to sprint performance. Therefore, the purpose of this study was to determine the relative effectiveness of TT and CT strength-power training models to improve sprint performance.

## Material and Methods

### Experimental Design

Two strength-power oriented training models (TT and CT) were performed over a 9-week period to determine their effectiveness to improve sprint performance. Total training volume was equated between TT and CT. During TT model, HRT, loaded JS, and CMJ were performed each in separate and successive three-week mesocycles. During the CT model, HRT, loaded JS, and CMJ were trained simultaneously for nine weeks.

The one-repetition maximum (1RM) smith-machine squat, CMJ height, 20-m sprint speed were assessed at baseline and weeks three, six and nine. Mean power (MP) and mean propulsive power (MPP) in the loaded jump squat (45% 1RM) were assessed pre- and post-training to quantify the changes in lower limb power production.

### Subjects

Forty-eight male soldiers of the Brazilian special operations brigade with a minimum of one year army training volunteered for this study. The subjects were divided into a control group (CG: n = 15, age: 20.2 ± 0.7 years, body height: 1.74 ± 0.06 m, and body mass: 66.7 ± 9.8 kg), a traditional training group (TT: n = 18, age: 20.1 ± 0.7 years, body height: 1.71 ± 0.05 m, and body mass: 64.2 ± 4.7 kg), and a complex training group (CT: n = 15, age: 20.3 ± 0.8 years, body height: 1.71 ± 0.07 m; and body mass: 64.0 ± 8.8 kg). The subjects followed a five-day on and two-day off routine in the army living quarters. All training groups were from the same military camp/company and performed all the daily tasks together. Thus, it was assumed that there were no nutritional and/or training routine differences between the three training groups. The subjects were instructed to refrain from all types of exercise/activities with the exception of the experimental training protocols and the regular army training (i.e. aerobic exercise, calisthenics, and strength-endurance circuit training) throughout the duration of the study. The subjects were informed of the experimental risks, and they signed an informed consent form before participation. An Institutional Review Board (Research Ethics Committee, CEP-EEFEUSP) for use of human subjects approved the investigation.

### Maximum strength testing

The 1RM was assessed as follows: the subjects ran for five minutes on a treadmill (Movement Technology, Brudden, São Paulo, Brazil) at 9 km·h^−^¹, followed by five minutes of lower limb active stretching exercises. Then, they executed two parallel smith-machine squat warm-up sets. In the first set, the subjects performed five repetitions with 50% of the estimated 1RM, and in the second set, they performed three repetitions with 70% of the estimated 1RM. A 3-minute rest interval was allowed between sets. Three minutes after the warm-up, the participants had up to five attempts to obtain the 1RM load (e.g., maximum weight that could be lifted once using proper technique), with a 5-minute interval between attempts. Strong verbal encouragement was given throughout the test (Brown and Weir, 2001) (within-subject coefficient of variation <5%).

### Countermovement jump height testing

Subjects were instructed to place their hands on their hips and freely determine the amplitude of the countermovement in order to avoid changes in jump coordination. They performed five jumps with a 15-second interval between attempts (within-subject coefficient of variation <10%). The jumps were performed on a contact platform (Winlaborat, Buenos Aires, Argentine) which measures flight time. The obtained flight time (t) was used to estimate the height of the body’s center of gravity (h) during the vertical jump (i.e., h = g·t^2^/8, where g = 9.81 m·s^−2^). The best attempt was used for data analysis.

### 20-m sprint testing

Two pairs of photocells (Winlaborat, Buenos Aires, Argentine) were used to mark a 20-m distance. The subjects accelerated for 5 meters before crossing the first pair of photocells (starting line) and were instructed to run as fast as possible for the next 20 meters (within-subject coefficient of variation <10%). They had 2 attempts, and the best one was considered for further analysis. The rest interval between the 2 attempts equaled 3 minutes.

### Loaded jump squat testing

During the loaded jump squat testing the subjects were instructed to start from a static squat position (i.e., ∼90° of knee flexion) and jump as high as possible without losing contact with the bar, using a load corresponding to 45% of the smith-machine squat 1RM. A linear transducer (T-force, Dynamic Measurement System, Ergotech Consulting S.L., Murcia, Spain) was attached to the Smith machine bar. Bar position data were sampled at a frequency of 1,000 Hz and recorded into a computer. Finite differentiation technique was used to estimate the derived mechanical variables. The bar displacement was obtained by integration of velocity (v) data with respect to the time; the acceleration was obtained from differentiation of velocity with respect to the time; the force (F) was calculated as F = m · (a + g), where m is the moving mass (kg) and g is the acceleration due to gravity; the power output resulted from the product of the vertical applied force and bar velocity (P = F · v). Mean power (MP) of each repetition was obtained by multiplying the average concentric force by the average concentric velocity during the entire concentric portion of the movement. Mean propulsive power was obtained by using the same method as before, but considering only the portion of the concentric phase during which the measured acceleration is greater than acceleration due to gravity (i.e., a ≥ − 9.81 m · s ^−2^) (Sanchez-Medina et al., 2010) (within-subject coefficient of variation <10%). They had 3 attempts, and the best one was considered for further analysis. We opted for using MP and MPP instead of using peak power as Sanchez-Medina et al. (2010) demonstrated that referring the mean mechanical values during the propulsive phase better reflected the differences in the neuromuscular potential between two given individuals. This approach avoids underestimation of true strength potential as the higher the mean velocity is (and lower the relative load), the greater is the relative contribution of the braking phase to the entire concentric time.

### Training protocols

The TT and the CT training protocols were comprised of a parallel smith-machine squat exercises, a loaded JS starting from approximately 90° of knee flexion, and a body mass CMJ with hands on the hips and self-adjusted countermovement amplitude. The total volume was equated across the training groups. Table 1 shows the training protocols for both groups over the 9-week period.

### Statistical analysis

Means and standard deviations (SD) were used to represent centrality and spread of the data; all variables were also assessed for normality (Shapiro-Wilk test). As the experimental groups were balanced and randomized based on smith-machine squat 1RM values, a one-way ANOVA was used to test for differences in the initial values between groups for all dependent variables. There were no differences in the initial values across all tested variables. A two-way repeated measures ANOVA with Tukey post hoc comparisons was used to determine if any significant differences existed between training groups across testing sections. The significance level was set at p≤0.05. To calculate the TEC between smith-machine squat 1RM, CMJ height, MP, MPP and the 20-m sprint performance, we used the equation proposed by Zatsiorsky and Kraemer (2006), as follows:

$$\begin{array}{l} TEC=\mathrm{Result}\;\mathrm{gain}\;(\mathrm{ES})\;\mathrm{in}\;\mathrm{unperformed}\;\mathrm{exercise}\\ /\mathrm{Result}\;\mathrm{gain}\;(\mathrm{ES})\;\mathrm{in}\;\mathrm{performed}\;\mathrm{exercise} \end{array}$$

TEC were only calculated between variables that had an ES of at least 0.35, which is related as the smallest difference to be considered when analyzing moderately trained subjects (Rhea, 2004).

## Results

**Figure 1:**
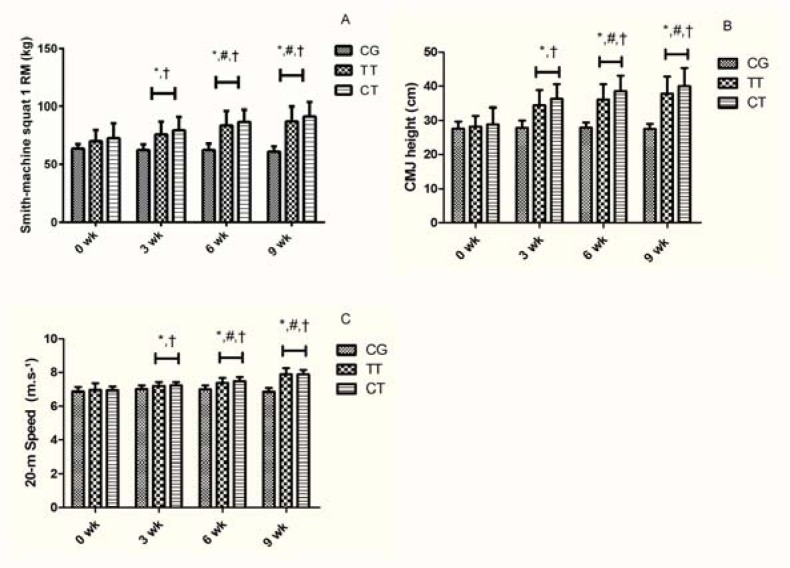
Maximum strength (smith-machine squat 1RM, kg – panel A), countermovement jump (CMJ) height (cm – panel B) and 20-m sprint speed (m·s^−1^ – panel C) pre- and post-training for the control (CG), traditional training (TT), and complex training (Chodzko-Zajko et al.) groups, at the instants 0-week (pre-training), 3-week, 6-week, and 9-week (post-training) (Mean ± SD). * - p≤0.05 compared to the pre-test values # - p≤0.05 compared to the previous time point † - p≤0.05 compared to the control group at the same time point

**Figure 2:**
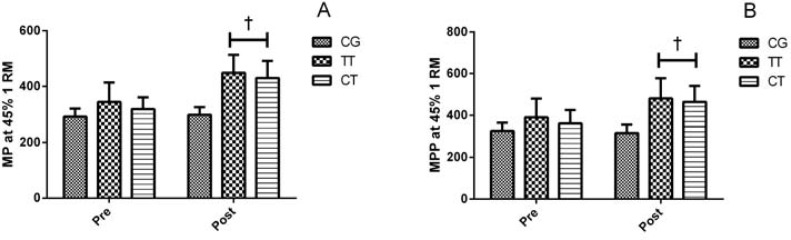
Mean power (MP-W, panel A) and mean propulsive power (MPP-W, panel B) in jump squat using a load of 45% 1RM, pre- and post-training for the control (CG), traditional training (TT), and complex training (CG) groups, at the instants 0-week (pre-test) and 9-week (post-test) (Mean ± SD). † - p≤0.05 compared to the control group at the same time point

The TT and CT significantly (p≤0.05) increased smith-machine squat 1RM (25% and 26%), CMJ height (36% and 39%) and 20-m sprint speed (16% and 14%), respectively. There were no significant differences (p>0.05) between training groups (Figure 1A, 1B and 1C). In addition, TT and CT groups produced significantly higher (p≤0.05) MP and MPP in the loaded JS in comparison to the CG following the training period (Figure 2A, 2B, and 2C).

Effect sizes and percentage increases for a non-trained variable (i.e. 20-m sprint speed) and all trained variables (i.e. RM, MP, MPP and CMJ) were greater in the TT group in comparison to the CT group following the training period (Table 2).

Table 3 shows the percentage ratio comparisons and TEC between the changes in a non-trained variable (i.e. 20-m sprint speed) and all trained variables (i.e. RM, MP, MPP and CMJ) after the experimental period. These variables were consistently higher for the TT group in comparison to the CT group following the 9-week training period.

## Discussion

Based on current findings, it is plausible to increase sprint performance through traditional and complex strength-power training in moderately trained subjects. It also appears that the TT had a greater transfer effect on sprint performance.

As previously mentioned, it has been advocated that the usage of CT is capable of maximizing the transfer effects from strength-power capacities to sprint performance due to a possible enhancement in neuromuscular activity (Young et al., 1998; Ebben, 2002; Docherty et al., 2004). However, some researchers have reported that CT only mirrors (e.g. 3–6 weeks) gains promoted by other training models, without being able to match the chronic adaptations (e.g. > 6 weeks) promoted by traditional strength-power training (Mihalik et al., 2008; MacDonald et al., 2012). TT and CT presented similar improvements in strength-power capabilities and sprint performance throughout the experimental period. The TEC presented by TT from all the measured variables (i.e., RM, MP, MPP and CMJ) to 20-m sprint performance were higher at 9-week time point (post-training) when compared to CT. However, based on the TEC, the strength and power developed through the TT had greater transference to 20-m sprint performance, in comparison to the strength and power developed through the CT. These findings refute the notion that CT provides a better stimulus for improved sprint performance.

This is the first study to compare TEC from strength-power training programs to sprint performance. Thus, the comparison of our data with the available literature was limited. Weiss et al. (2000) compared the TEC between two techniques of machine-based squat exercise (i.e., deep versus shallow) and different types of vertical jumps.

After 9 weeks, the authors concluded that deep machine-based squat training is preferable to the shallow exercise to increase muscle strength at any exercise depth. Moreover, the TEC for deep squats to both depth vertical jump (1.68) and restricted-motion standing vertical jump (2.32) was substantially greater than for shallow squats (0.11 and 0.31, respectively). It should be mentioned that TEC calculation may be biased since the measured variables were directly collected in the performed exercise, in contrast to the guidelines proposed by (Zatsiorsky et al., 2006).

Zatsiorsky’s coefficient of transfer (Zatsiorsky and Kraemer, 2006) is a valuable tool for assessing improvement in a physical ability (e.g. sprint performance) due to a non-specific training stimulus (e.g. strength or power training). The possibility of increasing sprinting speed through traditional and complex training methods is viable, based on the transference training effects reported herein. Since there was no change in the CG’s sprint performance, it can be inferred that the sprint performance gains observed in the TT and CT groups were a direct result of these respective strength and power regimens. Although several researchers have demonstrated that various models of strength and power training programs were able to increase sprinting speed, no study has attempted to calculate TEC values (McBride et al., 2002; Cormie et al., 2010; Loturco et al., 2013). The absence of this calculation in some investigations can be explained by the applied experimental procedures and the impossibility to control over and isolate the specific training stimulus (Chelly et al., 2009; Comfort et al., 2012; Marques et al., 2013).

## Discussion

Zatsiorsky’s coefficient of transfer (Zatsiorsky and Kraemer, 2006) is a valuable tool for assessing improvement in a physical ability (e.g. sprint performance) due to a non-specific training stimulus (e.g. strength or power training). The possibility of increasing sprinting speed through traditional and complex training methods is viable, based on the transference training effects reported herein. Since there was no change in the CG’s sprint performance, it can be inferred that the sprint performance gains observed in the TT and CT groups were a direct result of these respective strength and power regimens. Although several researchers have demonstrated that various models of strength and power training programs were able to increase sprinting speed, no study has attempted to calculate TEC values (McBride et al., 2002; Cormie et al., 2010; Loturco et al., 2013). The absence of this calculation in some investigations can be explained by the applied experimental procedures and the impossibility to control over and isolate the specific training stimulus (Chelly et al., 2009; Comfort et al., 2012; Marques et al., 2013).

The larger TEC observed in the TT group may be attributed to the proposed neuromuscular and morphological adaptations of strength training in moderately trained subjects. It is plausible that the TT could have maximized neuromuscular adaptations providing a “better strength foundation” prior to developing power and sprint abilities (Matveyev, 1972; 1977; Plisk et al., 2003; Issurin, 2008; 2010).

The inclusion of TEC and percent ratio calculations may prove beneficial for evaluating the transference effects of a non-specific training stimulus (e.g. strength and power training) on a specific untrained performance measure (e.g. sprinting speed). These calculations may provide greater clarity to the practitioner and athlete/subject as well as improve programming of strength-power training protocols.

## Conclusion

In summary, our data suggest that moderately trained individuals were able to improve their sprint ability through the use of different strength-power oriented training programs. However, when comparing TT and CT, the strength and power TEC in relation to sprint performance were greater in the TT regimen. Therefore, the transfer effects of increasing strength and power to improve sprint performance are possible in moderately trained subjects. In conclusion, coaches and practitioners alike should consider the acute and chronic effects of various strength and power loading schemes on sprint performance and other sport discipline specific performance measures.

## Figures and Tables

**Table 1:** Training protocols for the traditional training group (TT) and the complex training group (Chodzko-Zajko et al.) over the 9-week training period

**Traditional Training Group (TT)**								
**Week-1**	**Week-2**	**Week-3**	**Week-4**	**Week-5**	**Week-6**	**Week-7**	**Week-8**	**Week-9**
*Session 1–3*	*Session 4–6*	*Session 7–9*	*Session 10–12*	*Session 13–15*	*Session 16–18*	*Session 19–21*	*Session 22–24*	*Session 25–27*
**SMS**	**SMS**	**SMS**	**LJS**	**LJS**	**LJS**	**CMJ**	**CMJ**	**CMJ**
*Session 1*	*Session 4*	*Session 7*	*Session 10*	*Session 13*	*Session 16*	*Session 19*	*Session 22*	*Session 25*
^*^(3X8/50%RM)	(3X6/60%RM)	(3X5/70%RM)	(3X6/30%RM)	(3X5/45%RM)	(3X4/60%RM)	(3X4)	(3X6)	(3X8)
*Session 2*	*Session 5*	*Session 8*	*Session 11*	*Session 14*	*Session 17*	*Session 20*	*Session 23*	*Session 26*
(3X6/55%RM)	(3X5/65%RM)	(3X4/75%RM)	(3X6/30%RM)	(3X5/45%RM)	(3X4/60%RM)	(3X4)	(3X6)	(3X8)
*Session 3*	*Session 6*	*Session 9*	*Session 12*	*Session 15*	*Session 18*	*Session 21*	*Session 24*	*Session 27*
(3X5/60%RM)	(3X4/70%RM)	(3X3/80%RM)	(3X6/30%RM)	(3X5/45% RM)	(3X4/60%RM)	(3X4)	(3X6)	(3X8)

**Complex Training Group (CT)** (Chodzko–Zajko et al.)								
**Week-1**	**Week-2**	**Week-3**	**Week-4**	**Week-5**	**Week-6**	**Week-7**	**Week-8**	**Week-9**
*Session 1–3*	*Session 4–6*	*Session 7–9*	*Session 10–12*	*Session 13–15*	*Session 16–18*	*Session 19–21*	*Session 22–24*	*Session 25–27*
**SMS**	**SMS**	**SMS**	**SMS**	**SMS**	**SMS**	**SMS**	**SMS**	**SMS**
(1X8/50%RM)	(1X6X55%RM)	(1X5X60%RM)	(1X6/60%RM)	(1X5X65%RM)	(1X4X70%RM)	(1X5/70%RM)	(1X4X75%RM)	(1X3X80%RM)
**LJS**	**LJS**	**LJS**	**LJS**	**LJS**	**LJS**	**LJS**	**LJS**	**LJS**
(1X6/30%RM)	(1X6X30%RM)	(1X6X30%RM)	(1X5/45%RM)	(1X5X45%RM)	(1X5X45%RM)	(1X4/60%RM)	(1X4X60%RM)	(1X4X60%RM)
**CMJ**	**CMJ**	**CMJ**	**CMJ**	**CMJ**	**CMJ**	**CMJ**	**CMJ**	**CMJ**
(1X4)	(1X4)	(1X4)	(1X6)	(1X6)	(1X6)	(1X8)	(1X8)	(1X8)

Smith-machine squat (SMS), loaded jump squat (LJS) and countermovement jump (CMJ) represent the training exercises

* (Sets X Repetitions / percentage of the smith-machine squat 1RM)

**Table 2:** Effect size (ES) and percentage increases in 20-m sprint speed (SS), smith-machine squat 1RM (1RM), mean power (MP) and mean propulsive power (MPP) in jump squat using a load of 45% 1RM and CMJ height (CMJ) following 9 weeks of traditional (TT) and complex (Chodzko-Zajko et al.) strength and power training

Group	SS	ES_SS_	1RM	ES_1RM_	MP	ES_MP_	MPP	ES_MPP_	CMJ	ES_CMJ_
TT (n = 18)	16	4.22	25	1.76	30	1.27	22	1.72	36	3.40
CT (n = 15)	14	2.06	26	1.46	35	0.94	28	0.96	39	2.29

**Table 3:** Percentage ratio comparisons and transfer effect coefficients (TEC) between the changes 20-m sprint speed (SS) and smith-machine squat 1RM (1RM), mean power (MP) and mean propulsive power (MPP) in jump squat using a load of 45% 1RM and CMJ height (CMJ) due to 9 weeks of traditional (TT) and complex (Chodzko-Zajko et al.) strength and power training.

	Percentage ratio comparisons				Transfer effect coefficients			
Group	SS/1RM	SS/MP	SS/MPP	SS/CMJ	SS/1RM	SS/MP	SS/MPP	SS/CMJ
TT (n = 18)	0.64	0.53	0.73	0.44	2.40	3.32	2.45	1.24
CT (n = 15)	0.53	0.4	0.5	0.36	1.41	2.19	2.15	0.90
